# Curative endoscopic resection of giant esophageal dedifferentiated liposarcoma: a case report and literature review

**DOI:** 10.3389/fmed.2025.1662503

**Published:** 2025-08-26

**Authors:** Mingqing Liu, Huimei Wang, Nan Zhang, Hong Xu

**Affiliations:** Department of Gastroenterology and Endoscopy Center, The First Hospital of Jilin University, Changchun, China

**Keywords:** liposarcoma, dedifferentiated, esophageal, endoscopic resection, histopathological analysis

## Abstract

Esophageal dedifferentiated liposarcoma (DDLPS) is extremely rare. We report a case of esophageal dedifferentiated liposarcoma (DDLPS) measuring 12.5 × 3.0 × 2.8 cm in a 62-year-old male presenting with a one-year history of progressive dysphagia. Esophagogastroduodenoscopy and computed tomography showed a large pedunculated submucosal tumor arising from the esophageal entrance and extending intraluminally to 35 cm from the incisor teeth, partially obstructing the esophageal lumen. Curative endoscopic resection was successfully achieved using a novel technique employing an externally placed snare and nylon loop outside the endoscope, thereby avoiding traumatic surgical operation. Histopathologic examination showed that the giant tumor was composed of a differentiated lipomatous component adjacent to dedifferentiated spindle cells. Immunohistochemical analysis revealed spindle cells were positive for p16, CDK4, MDM2, CD34, and CD31. The differentiated lipomatous areas were positive for S-100. The definitive pathologic diagnosis confirmed a dedifferentiated liposarcoma, and the margin was negative. This represents the fifth reported case of esophageal DDLPS successfully managed through endoscopic resection. This externally deployed snare and nylon loop technique provides a viable and less invasive alternative for endoscopic resection of large pedunculated esophageal DDLPS. However, long-term follow-up is necessary to evaluate both therapeutic outcomes and prognosis fully.

## Introduction

Liposarcoma (LPS) is a rare malignant tumor that usually occurs in the retroperitoneum (50%) or extremities (25%) (1), and seldomly in the gastrointestinal tract (0.1–5.8% at autopsy) (2). It is scarce in the esophagus, representing 1.2–1.5% of all gastrointestinal liposarcomas (3). Based on pathological characteristics, liposarcoma is divided into four histologic subtypes: atypical lipomatous tumor/well-differentiated liposarcoma, dedifferentiated liposarcoma, myxoid liposarcoma, and pleomorphic liposarcoma. Most esophageal liposarcomas are well-differentiated liposarcomas (4), and primary esophageal dedifferentiated liposarcoma (DDLPS) has a very low incidence. Owing to its extreme rarity, standardized management protocols are lacking, and experience in the treatment of esophageal DDLPS is limited. Previously, surgical resection was the most common treatment for such lesions; however, in the management of complex giant esophageal liposarcomas, challenges such as the risk of bleeding and high recurrence rates persist (5, 6). Surgical resection persists as crucial for treating giant esophageal DDLPS; nonetheless, there is currently no standardized protocol for determining the appropriate surgical approach. Advances in endoscopic technology have facilitated a substantial shift toward minimally invasive approaches, representing a substantial progression in the management of this rare disease. Compared to surgery, endoscopic resection is less traumatic and more economical. We present a rare case demonstrating the successful application of a novel endoscopic technique utilizing an externally placed snare and nylon loop for the minimally invasive and curative resection of a giant esophageal DDLPS, effectively overcoming the limitations of standard endoscopic approaches for such large, high-location lesions.

## Case presentation

**Figure 1:**
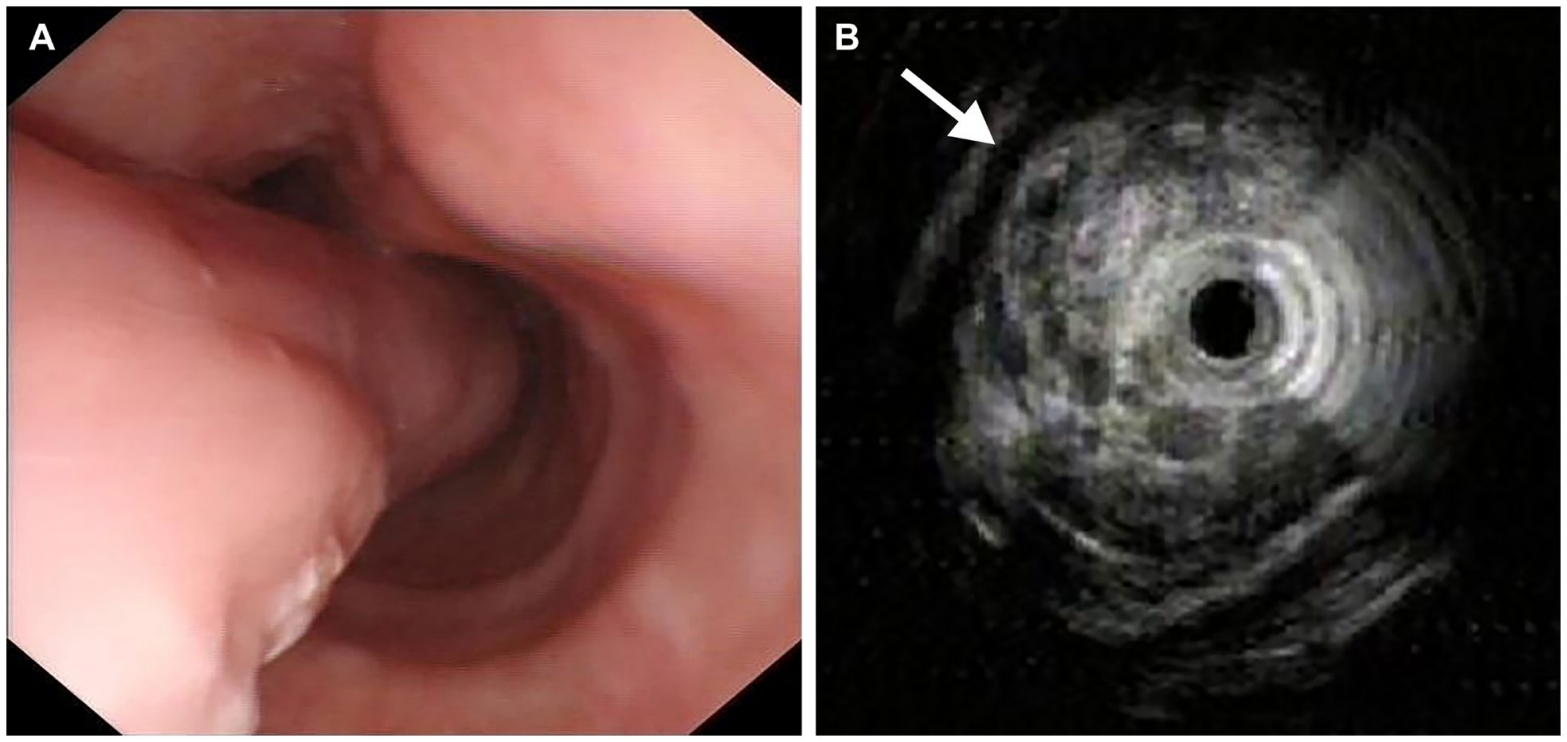
Endoscopic images. **(A)** Esophagogastroduodenoscopy revealed a giant pedunculated mass with a thick stalk, and the surface was covered with smooth mucous membrane. **(B)** Endoscopic ultrasonography showed a mixed-echogenicity with mid-to-high echo and hypervascular mass (arrow).

**Figure 2:**
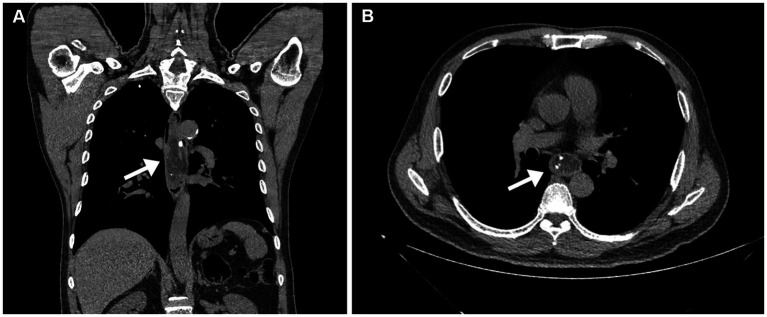
Computed tomography scan. **(A)** Computed tomography scan demonstrated esophageal dilation and a giant mass emanating from the esophageal entrance into the thoracic esophagus (arrow). **(B)** The mass appeared as an uneven, low-density, and nodular calcified shadow, with abundant blood flow (arrow).

On January 7, 2021, a 62-year-old man presented with dysphagia for more than 1 year without loss of weight. His medical history included 10 years of diabetes mellitus and 20 years of smoking. There was no obvious abnormality in the physical examination. All laboratory examinations were within the normal range. It is worth noting that there was an episode of vomiting followed by extrusion of a mass from the mouth 1 month ago. Then, the patient underwent esophagogastroduodenoscopy (EGD), which revealed a pedunculated mass with a 2.0 cm-sized stalk originating at the esophageal entrance and extending intraluminally to 35 cm from the incisor teeth. The head of the mass was about 5.0 cm long and 3.0 cm wide, and partially obstructed the esophageal lumen. The pedunculated mass exhibited a smooth surface without ulceration or erosion, and the mass texture was soft and easily deformed by pressure (Figure 1A). In order to examine the nature of the mass, we performed endoscopic ultrasonography (EUS), which demonstrated a mixed-echogenicity with mid-to-high echo and hypervascular mass (Figure 1B). Computed tomography (CT) scan demonstrated esophageal dilation and a giant intraluminal mass arising from the esophageal entrance and extending into the thoracic esophagus, and the head almost filled the lumen. The mass appeared as a low-density and nodular calcified shadow with abundant blood flow (Figures 2A,B). Based on the aforementioned results, it was suspected that the mass was a giant esophageal submucosal tumor derived from mesenchymal tissue, and no other lesions or enlarged lymph nodes were found.

**Figure 3:**
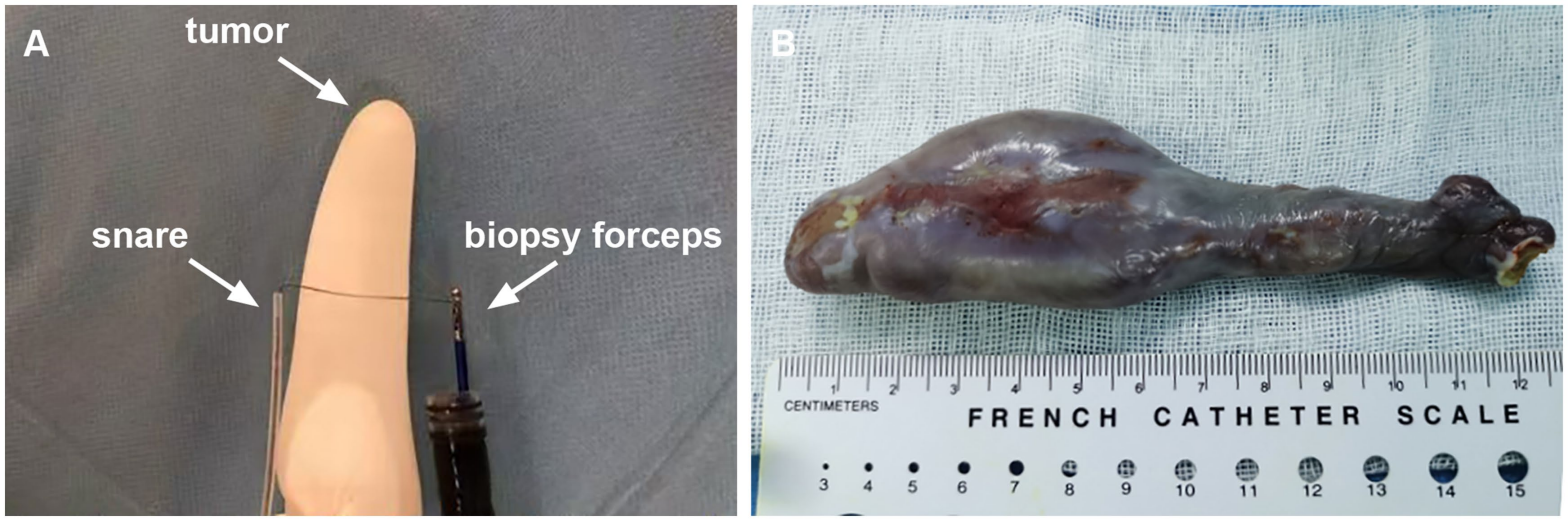
**(A)** The schematic diagram of endoscopic resection. **(B)** Macroscopic appearance of the giant esophageal submucosal tumor.

After multidisciplinary discussion involving gastroenterology, thoracic surgery, radiology, and oncology, endoscopic resection was selected over surgical intervention based on several key considerations. The patient’s comorbidities, including type 2 diabetes and a 20-pack-year smoking history, were associated with significantly elevated perioperative risks for major thoracic surgery. Lesion characteristics also favored endoscopic management: its pedunculated morphology with a stalk located at the esophageal inlet, along with entirely intraluminal localization and no evidence of deep invasion or extraluminal extension, lymph node or distant metastases on EUS and CT, suggested that complete resection was technically feasible. Despite its large size, the lesion’s mobility, soft consistency, and pedunculated configuration facilitated endoscopic manipulation. Compared to surgery, endoscopic resection offered substantially lower risks of complications such as nerve injury, anastomotic leakage, and infection, as well as shorter hospitalization and reduced overall costs. Meanwhile, the patient also expressed a preference for endoscopic resection as the treatment option. However, the traditional endoscopic operation posed significant challenges due to the narrow space, restricted visual field, and abundant blood vessels. To overcome the significant challenges, we proposed a novel endoscopic resection technique. This innovative approach involved introducing both the nylon loop and the snare externally alongside the endoscope, rather than through the instrument channel (Figure 3A). The lesion was ligated and subsequently resected by grasping the nylon loop or snare through biopsy forceps that had been inserted into the endoscopic biopsy channel. Initially, the nylon loop (maximum diameter 30 mm; Olympus Corporation, Japan) was gently attached to the distal end of the endoscope (Olympus GIF-Q260J; Olympus Corporation, Japan) and inserted into the esophagus alongside the endoscope. Subsequently, biopsy forceps (JHY-FB-23-160-O-P, JIUHONG, China) were advanced through the endoscope channel to grasp the distal end of the nylon loop. The endoscopist gradually adjusted the long axis of the nylon loop to be perpendicular to the long axis of the lesion, after which the endoscope, biopsy forceps, and nylon loop were simultaneously retracted. Leveraging the gravitational pull of the lesion, the torque transmitted via the biopsy forceps, and gentle rotation of the endoscope, the nylon loop successfully reached the base of the stalk. The endoscopist tightened the nylon loop and then released it to occlude the basal blood supply effectively. Within a few seconds, the lesion began to turn purple. Next, the snare (maximum diameter 35 mm; JIUHONG, China) was similarly inserted into the esophagus and positioned 1 cm away from the nylon loop to secure the root of the lesion. Using the forced coagulation mode (Effect 2, 40 W, VIO 200D, ERBE Germany), transection of the stalk was performed, achieving curative resection without complications. Additionally, the nylon loop contributed significantly to wound closure beyond its role in preventing intraoperative bleeding. The entire procedure lasted 50 min, with the endoscopic operation itself taking only 26 min (Supplementary Video S1).

**Figure 4:**
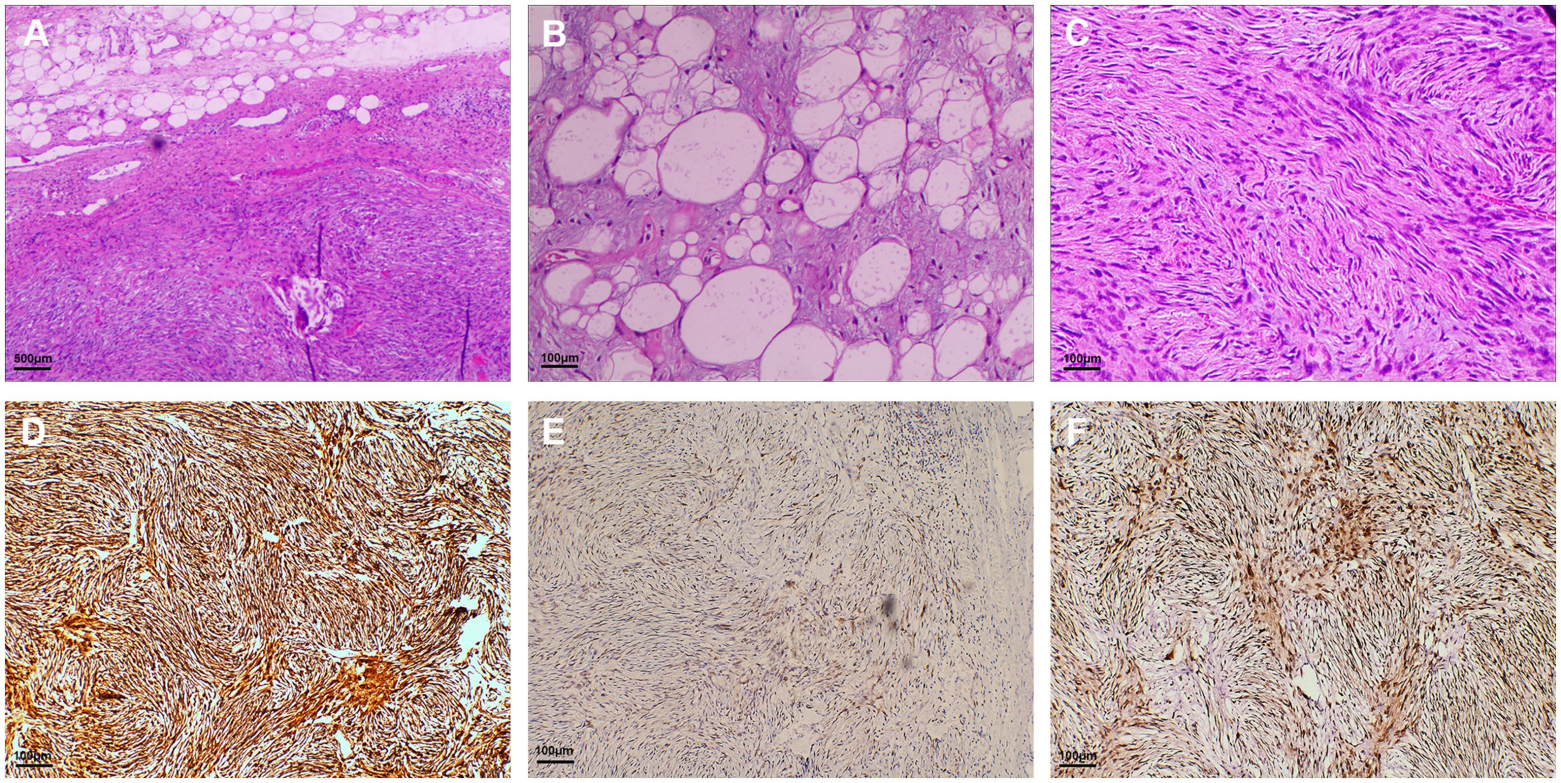
Hematoxylin and eosin-stained and immunohistochemical staining sections. **(A)** Histopathologic examination showed that the giant tumor was composed of differentiated lipomatous component and dedifferentiated spindle cell sarcomaedifferentiated component of the liposarcoma (magnification × 40); **(B)** Examination of the differentiated lipomatous areas showed adipose tissue with fibrous septa (magnification × 200); **(C)** The dedifferentiated component of the hypercellular spindle cells without lipogenic differentiation (magnification × 200). Immunohistochemical analysis revealed spindle cells were positive for p16 **(D)**, CDK4 **(E)**, and MDM2 **(F)** (magnification × 200).

Macroscopically, the giant pedunculated mass was measured 12.5 × 3.0 × 2.8 cm with a thick stalk and a spindle-like head (Figure 3B). Histopathologic examination (Figure 4) showed that the huge tumor was composed of a differentiated lipomatous component adjacent to dedifferentiated spindle cells (Figure 4A). Examination of the differentiated lipomatous areas showed adipose tissue with fibrous septae containing scattered lipoblasts, atypical hyperchromatic stromal cells, mucoid degeneration, and ossification (Figure 4B). The dedifferentiated spindle cells showed marked cytological atypia, nuclear hyperchromasia, and brisk mitotic activity (Figure 4C). Immunohistochemical analysis revealed spindle cells were positive for p16 (Figure 4D), CDK4 (Figure 4E), MDM2 (Figure 4F), CD34, CD31. The differentiated lipomatous areas were positive for S-100. The definitive pathologic diagnosis confirmed a dedifferentiated liposarcoma, and the margin was negative.

The postoperative course was uneventful, and on the second day after the procedure, EGD showed that the nylon loop was in place and the wound healed well. We recommended that the patient undergo postoperative adjuvant treatment, but he refused and required only close follow-up. One month later, there were no signs of dysphagia, and the patient remained asymptomatic. EGD showed that the wound had healed and the surface was smooth. We recommend that the patient undergo rigorous clinical follow-up, including detailed history taking and physical examinations every 3 months. The follow-up interval may be extended to every 6 months after the first 3 years. A follow-up chest CT and EGD are recommended within 6 months for the first 3 years, and once a year thereafter. There was no recurrence until January 2025.

## Literature review

A search in PubMed was carried out using the following query string: (“Esophagus” [Mesh] OR esophagus OR esophageal OR esophageal) AND (Liposarcoma, Dedifferentiated OR dedifferentiated liposarcoma OR DDLPS OR high-grade liposarcoma) OR (“Esophageal Neoplasms” [MeSH], “Esophageal Diseases” [MeSH]). The relevant articles found are shown in Table 1. Ultimately, a total of 14 cases of DDLPSs were retrieved, among which 4 cases were treated endoscopically, 6 cases underwent surgical treatment, 1 case received chemotherapy, and 9 cases showed no recurrence during postoperative follow-up (6–17). Based on the cases, esophageal DDLPS tends to present in middle-aged to elderly male patients. The average age was 61.7 years, and the male and female ratio was 11:3. These tumors have the potential to grow to large sizes, and reported tumor sizes ranged from 5 cm to a maximum length of 20 cm.

**Table 1:** Demographics, clinical presentation, lesion characteristics, treatment, and follow-up of dedifferentiated liposarcoma (DDL) of the esophagus.

Author	Year of publication	Age (year)	Gender	Symptom	Type of lesion	Location of lesion initiation	Tumor size (cm)	Treatment	Adjuvant therapy	Follow-up
Parikh et al. (13)	2019	58	M	Dysphagia	Polypoid	Cervical	18	Endoscopic resection	No	Not mentioned
Brett et al. (10)	2016	75	M	Dysphagia	Polypoid	Cervical	5.0 × 2.0 × 2.8	Endoscopic resection	No	No recurrence at 20 months
Torres-Mora et al. (9)	2012	81	M	Dysphagia	Polypoid	Cervical	7.3 × 2.8 × 1.4, 4.5 × 2.8 × 1.2	Endoscopic resection	Not mentioned	No recurrence at 1 month
Will et al. (7)	2007	60	M	Dysphagia	Polypoid	Cervical	20 × 4 × 4	Endoscopic resection	Not mentioned	No recurrence at 12 months
Omachi et al. (17)	2024	69	M	Not mentioned	Polypoid	Cervical	9.5 × 4.0 × 2.3	Cervical oesophagotomy	Not mentioned	No recurrence at 12 months
Pham et al. (16)	2022	76	F	Dysphagia, weight loss	Sessile	Lower	6 × 7	Chemotherapy	Not mentioned	Not mentioned
Ng et al. (6)	2019	54	M	Palpitation, exertional dyspnoea	Polypoid	Cervical	14.4 × 5.8	Cervical oesophagostomy	Not mentioned	No recurrence
Shi et al. (14)	2020	38	F	Swallowing obstruction, dysphagia, pharyngalgia	Polypoid	Cervical	12.5 × 6.5 × 2.5	Surgery	Not mentioned	No recurrence at 6 months
Ye et al. (15)	2020	38	F	Dysphagia, dyspnea	Polypoid	Cervical	20	Thoracoscopic surgery	No	No recurrence at 12 months
Graham et al. (12)	2018	67	M	Not mentioned	Polypoid	Distal	5.2	Not mentioned	Not mentioned	Died at 37 months
Graham et al. (12)	2018	42	M	Not mentioned	Polypoid	Proximal	10	Not mentioned	Not mentioned	Alive at 29 months
Graham et al. (12)	2018	75	M	Not mentioned	Polypoid	Proximal	Not mentioned	Not mentioned	Not mentioned	Alive at 29 months
Riva et al. (11)	2016	81	M	Dysphagia, weight loss	Polypoid	Cervical	21 × 8 × 3.5	Cervical oesophagotomy	No	No recurrence at 12 months
Watkin et al. (8)	2011	50	M	Dysphagia, weight loss, cough, dyspnea	Polypoid	Lower	10 × 8 × 6	Subtotal oesophagectomy	radiotherapy	No recurrence at 51 months

## Discussion

Liposarcoma arises from the mesenchymal layer and predominantly affects the retroperitoneum, trunk, and extremities, with rare occurrences in the esophagus (18). Mansour et al. described the first report of a primary esophageal liposarcoma in 1983 (19). As we know, four main pathologic subtypes of liposarcoma are described, and esophageal DDLPS is a rare type with a prevalence of approximately 6% of all liposarcomas (2, 4, 18, 20). In this report, we present a case of a large primary esophageal DDLPS that was successfully managed using a novel endoscopic resection technique.

Esophageal DDLPS typically behaves as a slow-growing tumor, and patients usually present with progressive dysphagia, weight loss, dyspnea, or throat discomfort (17). It is worth noting that a pedunculated liposarcoma can be quite mobile in the esophageal lumen and even reach into the stomach or prolapse into the mouth. It may cause sudden death from life-threatening asphyxia due to causing laryngeal obstruction by prolapse into the mouth. Just like this case, the patient was aware of a mass prolapse into the mouth after vomiting. Therefore, during the early endoscopic examination, we should adopt unsedated endoscopy, tracheal intubation, or semi-recumbent position to avoid asphyxia.

During the initial endoscopic evaluation, biopsies were not performed. The decision to forgo biopsy was based on several key factors. First, while endoscopic ultrasound-guided fine-needle aspiration/biopsy (EUS-FNA/B) is recommended for the evaluation of such lesions, its diagnostic accuracy for subepithelial tumors is known to be highly variable, with reported success rates ranging from 20 to 93% in the literature (21, 22). Second, according to established guidelines and published evidence on the application of EUS-FNA/B, preoperative histopathological confirmation is not considered mandatory for subepithelial tumors that are deemed resectable (23, 24). Finally, given the lesion’s large size and hypervascular appearance on EUS and CT, we determined that biopsy might carry a significant risk of complications, including bleeding or infection. Following a comprehensive discussion outlining the risks associated with biopsy compared to the planned definitive resection, the patient clearly expressed a preference to proceed directly to curative resection and obtain a definitive diagnosis through postoperative pathological analysis. The multidisciplinary team subsequently endorsed this management strategy after a consensus discussion.

The therapeutic principle of esophageal liposarcoma is resection with clear margins. Surgical excision has classically been the optimal treatment method; however, surgical approaches are expensive and invasive. In recent years, with advances in endoscopic technology, endoscopic resection has become an alternative option for esophageal liposarcoma (6). We conducted a retrospective analysis to evaluate the demographic, clinical, and pathological characteristics of 20 patients with esophageal liposarcoma who underwent endoscopic resection (2, 7, 9, 10, 13, 14, 25–38), with the results summarized in Supplementary Table S1. Our case represents the 21st reported case of esophageal liposarcoma treated with endoscopic resection. We reviewed the relevant literature and summarized that endoscopic resection was preferred to surgery for esophageal liposarcoma for the following reasons (6, 7, 36): (a) intraluminal pedunculated lesion, (b) the lesion did not involve the deep layers of the esophageal wall, (c) CT indicated no lymph node or distant metastasis, and (d) there was sufficient space within the esophageal lumen to perform curative endoscopic resection. By summarizing the cases of endoscopic treatment of liposarcoma, it is found that endoscopic resection can achieve negative margins, effectively prevent bleeding and other complications, and has less trauma, faster recovery, and lower cost compared with surgical resection. However, it is indeed necessary to closely follow up these cases to observe the long-term efficacy of endoscopic resection. At the same time, for extremely rare esophageal liposarcoma, a multidisciplinary consultation is still needed to assess and select the best treatment plan fully.

The majority of esophageal dedifferentiated liposarcomas commonly arise from the cervical and upper esophagus, presenting as intramural submucosal pedunculated lesions with long vascularized pedicles covered by normal mucosa. These lesions usually grow to large sizes, encompassing the esophageal lumen (27). Therefore, due to the large size, abundant blood flow, high location, and small space, the traditional endoscopic resection method, such as endoscopic mucosal resection (EMR) or endoscopic submucosal dissection (ESD), is challenging to operate in a small space to achieve complete resection. Endoscopic piecemeal mucosal resection (EPMR) would damage the integrity of the lesion and increase the risk of bleeding. The core innovation of our technique lies in the external placement of both the nylon loop and the snare alongside the endoscope. This fundamental departure from conventional endoscopic practice, where instruments are deployed through the working channel, provided several critical advantages for managing this giant, high-location, pedunculated tumor: (a) Preserved working channel function, enabling continuous endoscopic tool use (biopsy, irrigation) without the need to exchange instruments clogging the channel; (b) Enhanced maneuverability and precision by acting as a “third hand,” allowing independent, precise manipulation of loop or snare around the large lesion base in confined space; (c) Facilitated curative *en-bloc* resection via direct-vision positioning and securing of devices at the stalk base, preserving pathological integrity; (d) Overcame severe space constraints from the narrow lumen and tumor bulk, limitations that hinder traditional channel-deployed techniques. Therefore, when the snare is not feasible due to the difficulty of trapping the stalk or poses a risk of incomplete resection, creating a “third hand” assistance is a reliable option that is safe in patients with giant esophageal DDLPS. To our knowledge, this represents the first detailed description and successful application of this specific external instrument placement technique for the resection of a giant esophageal DDLPS.

There are some other lesions, such as lipoma, fibroepithelial polyp, gastrointestinal stromal tumor, leiomyoma, leiomyosarcoma, and other subtypes of liposarcoma in the esophagus, clinically and microscopically mimicking dedifferentiated liposarcoma, which makes it difficult to diagnose for clinicians and pathologists (5). EGD images and radiology diagnostic modalities such as barium swallow, CT, or magnetic resonance imaging (MRI) features are both nonspecific, and definitive diagnosis of DDLPS can only be achieved by histological examination. The genetic hallmark of DDLPS is giant or supernumerary ring chromosomes that contain eminent amplification of chromosome 12q14-15. This area of chromosome 12 includes MDM2, CPM, HMGA2, CDK4, and SAS/TSPAN31, with the MDM2 gene being considered the primary driver of DDLPS (39). Also, high amplification levels of MDM2 correlate with poor outcomes in patients with dedifferentiated liposarcoma (14). A previous study showed the immunohistochemical trio of CDK4, MDM2, and p16 is highly sensitive and specific in the differential diagnosis of DDLPS (40). Confirmation of MDM2 gene amplification is considered to be the gold standard, and molecular analysis such as fluorescence *in situ* hybridization (FISH), quantitative PCR, and comparative genomic hybridization can be applied to provide a reliable diagnosis. However, MDM2 gene amplification is not unique in DDLPS, and the presence of MDM2 gene amplification cannot be equated with the presence of a DDLPS (41). Therefore, the diagnosis of DDLPS should be confirmed by a series of results, including hematoxylin–eosin (HE) stain, immunohistochemistry, and molecular analysis.

Compared to other pathologic subtypes, the DDLPS has a higher local recurrence rate (41%), distal metastatic rate (17%), and disease-related mortality rate (28%) (42). A positive resection margin is associated with local recurrence and metastasis. Previous studies showed DDLPS was relatively chemoresistant, and postoperative adjuvant chemotherapy was rarely employed for localized DDLPS. However, DDLPS is moderately sensitive to radiation, and radiation is applied in the case of DDLS of the extremity and retroperitoneum to reduce the risk of local recurrence (43). Watkin et al. (8) described a case of a 50-year-old male with DDLPS who underwent subtotal esogastrectomy and postoperative radiotherapy and was free of disease for 4 years and 3 months. However, the postoperative adjuvant chemotherapy and radiation therapy experience, and long-term follow-up are insufficient; further studies are needed to standardize the treatment plan. In addition, there are no biomarkers available for the postoperative monitoring of patients. However, regular EGD, radiographic surveillance, and physical examination remain the main contents of follow-up. It is expected that, through more in-depth research on DDLPS in the future, specific biomarkers can be obtained for postoperative monitoring (13).

## Conclusion

Esophageal DDLPS is an exceedingly rare tumor, and curative resection is still the mainstay of treatment. This represents the fifth reported case of esophageal DDLPS successfully managed endoscopically and, crucially, the first utilizing our novel technique of externally placing both the occluding nylon loop and the resection snare alongside the endoscope. This innovative approach provides a viable, less invasive, and effective alternative to surgery for the endoscopic management of large, challenging pedunculated esophageal DDLPS, particularly where conventional endoscopic techniques are limited by size, location, or space constraints. However, long-term follow-up is necessary to evaluate both therapeutic outcomes and prognosis fully.

## Data Availability

The data analyzed in this study is subject to the following licenses/restrictions: The original contributions presented in the study are included in the article, further inquiries can be directed to the corresponding author. Requests to access these datasets should be directed to x_hong@jlu.edu.cn.
