# Supplementary material for: Curative endoscopic resection of giant esophageal dedifferentiated liposarcoma: a case report and literature review
Source: Front Med (Lausanne). 2025 Aug 26;12:1662503. doi: 10.3389/fmed.2025.1662503 (PMC12417198; doi:10.3389/fmed.2025.1662503)
Supplement: Supplementary file 1 [file Table_1.docx]

**Supplementary Table S1. Demographics, clinical presentation, lesion characteristics, treatment, and follow-up of esophageal liposarcoma treated by endoscopic resection**

| **Author** | **Year of publication** | **Age (year)** | **Gender** | **Symptom** | **Type of lesion** | **Tumor size (cm)** | **Location of lesion initiation** | **Diagnosis** | **Treatment** | **Follow-up** | **Complications** | **Margin** |
| --- | --- | --- | --- | --- | --- | --- | --- | --- | --- | --- | --- | --- |
| Huang et al. (31) | 2025 | 54 | M | Dysphagia | Polypoid | 15 × 4.5 | Cervical | Well-differentiated | Endoscopic resection (ESD) | Not mentioned | No | Not mentioned |
| Ma et al.(33) | 2025 | 62 | M | Dysphagia | Polypoid | 22 × 4 × 4 | Cervical | Well-differentiated | Endoscopic resection (ESD) | No recurrence at 1 month | No | Not mentioned |
| Ma et al.(33) | 2025 | 41 | M | Dysphagia, weight loss | Sessile | 20 × 6 × 3 | Cervical | Well-differentiated | Endoscopic resection (ESD) | No recurrence at 1 month | No | Not mentioned |
| Wahba et al.(38) | 2025 | 64 | M | Dysphagia, bleeding | Polypoid | 30 | Cervical | Well-differentiated | Endoscopic resection and gastrotomy | No recurrence at 3 months | Not mentioned | Not mentioned |
| Lee et al.(32) | 2024 | 51 | M | Dysphagia | Polypoid | 8.3 × 4.2 × 2.3 | Cervical | Well-differentiated | Endoscopic resection (ESD) | No recurrence at 2 months | No | Not mentioned |
| Qi et al.(35) | 2024 | 56 | F | Extrusion of the mass | Polypoid | 14.0 × 3.0 × 3.0 | Cervical | Well-differentiated | Endoscopic resection (ESD) | No recurrence at 6 months | No | Not mentioned |
| Boghani et al.(28) | 2023 | 61 | F | Dysphagia | Polypoid | 5.0 × 0.7 × 0.4 | Cervical | Well-differentiated | Endoscopic resection, CO2 laser, balloon dilation | No recurrence at 12 months | Esophageal stenosis | Negative |
| Duan et al.(30) | 2023 | 69 | M | Dysphagia | Polypoid | 22 | Cervical | Well-differentiated | Endoscopic resection (ESD) | Not mentioned | No | Not mentioned |
| Parikh et al.(10) | 2019 | 58 | M | Dysphagia | Polypoid | 18 | Cervical | Dedifferentiated liposarcoma | Endoscopic resection | Not mentioned | No | Not mentioned |
| Cai et al.(29) | 2017 | 35 | M | Dull pain after food intake | Polypoid | 16.0 × 5.5 × 4 | Cervical | Well-differentiated | Endoscopic resection (ESD) | No recurrence at 4 years | Not mentioned | Not mentioned |
| Brett et al.(7) | 2016 | 75 | M | Dysphagia | Polypoid | 5.0 × 2.0 × 2.8 | Cervical | Dedifferentiated liposarcoma | Endoscopic (piecemeal) | No recurrence at 20 months | Not mentioned | Negative |
| Takiguchi et al.(2) | 2016 | 73 | M | Respiratory distress | Polypoid | 27.5 × 11.6 | Cervical | Well-differentiated | Endoscopic resection (ESD), oesophagotomy | No recurrence at 41 months | No | Negative |
| Valiuddin et al.(37) | 2016 | 68 | M | Dysphagia, retrosternal pain | Polypoid | 13.0 × 6.0 × 2.6 | Cervical | Rhabdomyomatous well-differentiated esophageal  liposarcomas | Endoscopic resection (snare and diathermy) | No recurrence at 4 years | Not mentioned | Negative |
| Yo et al.(39) | 2013 | 44 | M | Dysphagia | Polypoid | 8.7 × 6.0 × 3.0 | Cervical | Well-differentiated | Endoscopic resection (ESD) | No recurrence at 4 months | Not mentioned | Not mentioned |
| Aloraini et al. (26) | 2012 | 63 | M | Dysphagia | Polypoid | 4 × 2 × 2 | Cervical | Well-differentiated | Endoscopic resection | No recurrence at 6 months | No | Negative |
| Torres-Mora et al.(14) | 2012 | 81 | M | Dysphagia | Polypoid | 7.3 × 2.8 × 1.4, 4.5 × 2.8 × 1.2 | Cervical | Dedifferentiated liposarcoma | Endoscopic resection | No recurrence at 1 month | Not mentioned | Positive |
| Will et al.(16) | 2007 | 60 | M | Dysphagia | Polypoid | 4 × 4 × 20 | Cervical | Dedifferentiated liposarcoma | Endoscopic resection | No recurrence at 12 months | Bleeding | Not mentioned |
| Beaudoin et al.(27) | 2002 | 68 | F | Dysphagia, vomiting | Polypoid | 8.5 × 4.5 × 3 | Cervical | Well-differentiated | Endoscopic resection | Not mentioned | Not mentioned | Positive |
| Temes et al.(36) | 1998 | 69 | M | Dysphagia | Polypoid | 12 | Cervical | Well-differentiated | Endoscopic resection (Suture ligation) | Not mentioned | Not mentioned | Not mentioned |
| Masumori et al.(34) | 1991 | 46 | F | Protruding tumour in mouth | Polypoid | 11 × 4 × 3 | Cervical | Well-differentiated | Endoscopic resection | Not mentioned | Not mentioned | Not mentioned |

*ESD, endoscopic submucosal dissection*
